# Effects of Lipid Shape and Interactions on the Conformation, Dynamics, and Curvature of Ultrasound-Responsive Liposomes

**DOI:** 10.3390/pharmaceutics14071512

**Published:** 2022-07-21

**Authors:** Hwankyu Lee, Hyungwon Moon, Hyun-Ryoung Kim

**Affiliations:** 1 Department of Chemical Engineering, Dankook University, Yongin-si 16890, Korea; 2 R&D Center, IMGT Co., Ltd., 172, Dolma-ro, Bundang-gu, Seongnam-si 13605, Korea; hyungwon.moon@nanoimgt.com

**Keywords:** liposome, drug delivery, molecular dynamics simulation, ultrasound

## Abstract

We perform coarse-grained molecular dynamics simulations of bilayers composed of various lipids and cholesterol at their different ratios. Simulations show that cholesterol-lipid interactions restrict the lateral dynamics of bilayers but also promote bilayer curvature, indicating that these opposite effects simultaneously occur and thus cannot significantly influence bilayer stability. In contrast, lyso-lipids effectively pack the vacancy in the bilayer composed of cone-shaped lipids and thus reduce bilayer dynamics and curvature, showing that bilayers are more significantly stabilized by lyso-lipids than by cholesterol, in agreement with experiments. In particular, the bilayer composed of cone-shaped lipids shows higher dynamics and curvature than does the bilayer composed of cylindrical-shaped lipids. To mimic ultrasound, a high external pressure was applied in the direction of bilayer normal, showing the formation of small pores that are surrounded by hydrophilic lipid headgroups, which can allow the release of drug molecules encapsulated into the liposome. These findings help to explain experimental observations regarding that liposomes are more significantly stabilized by lyso-lipids than by cholesterol, and that the liposome with cone-shaped lipids more effectively releases drug molecules upon applying ultrasound than does the liposome with cylindrical-shaped lipids.

## 1. Introduction

Liposomes, which are artificial vesicles mainly composed of natural or synthetic phospholipids, have been widely used for drug delivery and antitumor therapeutics because they are biocompatible, soluble, and easy to be controlled and modified with functional groups [1,2,3,4]. Drug molecules can be encapsulated into the aqueous core of liposome and delivered to specific cancer or targeted cells [5,6,7,8,9]. To achieve this, liposomes need to remain stable for enough circulating lifetime in the bloodstream but also should become unstable to release drug molecules upon applying external stimuli [10,11,12]. To optimize these factors, liposomes composed of various lipid components with their different ratios have been characterized, and external stimulus methodologies such as temperature change (hyperthermia), light (photodynamic therapy), magnetic field, and pulse (ultrasound) have been applied [13,14,15,16,17,18,19,20]. In particular, ultrasound wave is clinically safe, inexpensive, and portable and thus has been considered a promising physical stimulus for drug delivery applications [21,22].

Since Zasadzinski found that ultrasonication significantly influences the structure and stability of liposomes [23], many experiments have shown that ultrasound can effectively trigger the release of drug molecules from liposomes in vivo and in vitro [24,25,26,27,28,29,30,31]. To optimize lipid components and ratios, Needham and coworkers characterized the stability, phase transition and drug-release rate of liposomes composed of various lipids, polymers, and cholesterol at different ratios, showing the temperature-sensitive liposomes that are stable at 37 °C but become unstable at 42 °C, leading to a rapid release of drug [32,33,34,35,36,37]. These experiments have revealed great potential of ultrasound for drug delivery applications and the effects of lipid components and ratios on the temperature-dependent phase behavior of liposomes, although the stability, phase transition, and drug-release rate of ultrasound-responsive liposomes have not yet been systematically examined. Recently, Kim et al. developed the ultrasound-responsive drug-loaded liposome by optimizing lipid components and ratios [38], called “IMP301”, showing the higher stability than do other commercial liposomes but also a sufficient release of drug molecules when applying ultrasound wave [39], which has been interpreted as the pore formation of liposome membrane caused by the cone-shaped lipid-induced negative curvature, although the effect of different lipids and the mechanism of drug release have not yet been well investigated because of the limited resolution of experimental techniques. To explore this, the effects of lipid components and ratios on the bilayer conformation, dynamics, and stability need to be studied at nearly the atomic scale, as can be done using molecular dynamics (MD) simulations.

In this work, we therefore perform coarse-grained (CG) MD simulations of bilayers composed of lipids and cholesterol at different ratios. To compare the conformation and stability of different bilayers, mass densities, lateral dynamics and the extent of curvature of bilayers were analyzed, which were favorably compared with experiments and rationalized by the lipid shape and lipid-lipid (or cholesterol) interactions. To mimic ultrasound wave, a high external pressure was applied in the direction of bilayer normal, showing the formation of pores that allow water molecules across the bilayer. We will show that these results help to explain in detail the experimental observations regarding the effects of specific lipids on the stability and drug-release rate of liposomes.

## 2. Materials and Methods

All simulations and analyses were performed using the GROMACS-2018.6 simulation package [40,41,42]. Potential parameters for lipids, cholesterol, and polyethylene glycol-grafted lipid (PEGylated lipid) were taken directly from the “MARTINI” CG force field (FF) [43,44], which lumps a few (three or four) heavy atoms into each CG bead. For PEGylated lipid, CG models were previously parameterized within the framework of the MARTINI CG FF by our group [45,46], which have successfully captured experimental results and polymer theories such as phase behaviors of self-assembled PEGylated lipids [47], the adsorption of plasma proteins onto PEGylated bilayers [48], and the mushroom-brush transition of PEG chains grafted to lipid bilayers and various nanoparticles [49,50]. A temperature of 290 K and a pressure of 1 bar were maintained by applying the velocity-rescale thermostat [51], and the Parrinello-Rahman barostat in an NP_xy_P_z_T ensemble (with semi-isotropic pressure coupling) [52]. Note that here we used a temperature of 290 K instead of the experimental temperature of 310 K [39] because the transition temperature of the CG DPPC bilayer between the rippled gel phase and liquid-crystalline phase is 295 K [53], which is much lower than the experimental transition temperature of 315 K [54]. Although the DPPC bilayer was not simulated in this work, the experimental temperature of 310 K is lower than the transition temperature of the DPPC bilayer (315 K), we used 290 K, which is lower than the transition temperature of the CG DPPC bilayer (295 K). To mimic ultrasound pressure, an external pressure of 100 bar was applied to the xy-plane of bilayer in the direction of bilayer normal. A real space cutoff of 1.2 nm was used for Lennard-Jones and Coulomb potentials with a smooth shift to 0 between 0.9 and 1.2 nm and between 0 and 1.2 nm, respectively. The LINCS algorithm was used to constrain the bond lengths [55,56]. Simulations were performed for 20 µs with a time step of 20 fs on computational facilities supported by the National Supercomputing Center with supercomputing resources including technical support (KSC-2021-RND-067).

### 2.1. Simulations of Bilayers at Different Lipid Ratios

**Figure 1:**
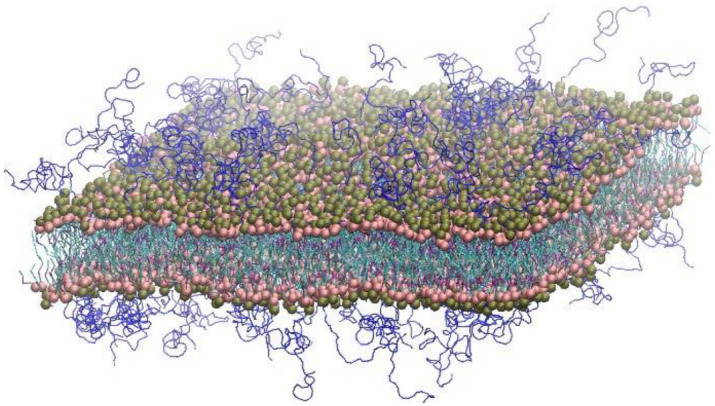
A snapshot of a IMP301 bilayer. Phosphate and choline (or ethanolamine) headgroups, hydrocarbon tails, PEG chains, and cholesterol are colored in pink, brown, light-blue, dark-blue, and purple, respectively. For clarity, water and ions are omitted. The images were created with Visual Molecular Dynamics [57].

Lipid bilayers, which consist of 1,2-distearoyl-sn-glycero-3-phosphorylcholine (DSPC), 1,2-distearoyl-sn-glycero-3-phosphoethanolamine with conjugated methoxyl poly(ethylene glycol2000) (DSPE-PEG), 1,2-dioleoyl-sn-glycero-3-phosphoethanolamine (DOPE), 1,2-dioleoyl-sn-glycero-3-phosphocholine (DOPC), 1-stearoyl-2-hydroxy-sn-glycero-3-phosphocholine (MSPC), and cholesterol at different lipid ratios as also used in experiments [38,39], were solvated with ~70,000 or ~280,000 water beads in a periodic box of size 27 × 27 × 16 nm^3^ or 54 × 54 × 16 nm^3^ (Table 1 and Figure 1). Since DSPE-PEG has a net charge of -1 per chain, counterions (128 Na^+^) were added to achieve electro-neutrality of bilayer systems.

### 2.2. Simulations of a DOXIL Bilayer

A lipid bilayer, which consists of hydro Soy PC (HSPC), cholesterol, and DSPE-PEG at molar ratios of 56:38:5 as also used in experiments [39], was solvated with ~76,000 or ~304,000 water beads in a periodic box of size 28 × 28 × 16 nm^3^ or 56 × 56 × 16 nm^3^. Counterions (196 Na^+^) were added to achieve electro-neutrality of the bilayer system. 

### 2.3. Calculation of the Contour Bilayer-Surface Area

The bilayer surface plane (x,y-plane of the bilayer system) was equally divided into 256 voxels (16 × 16 grids), leading to an x,y area of approximately 1.7 × 1.7 nm^2^ for each voxel with a voxel height (z component) of ~16 nm. For each voxel, the average x,y,z-coordinate was determined from the center of mass of glycerol beads in the upper leaflet of the bilayer, leading to a total of 256 coordinates in the bilayer surface. Bilayer edges need to be considered, and hence an additional 17th grid was generated using periodic boundary conditions, leading to a total of 289 coordinates. From those 289 points, 512 triangles were built, and their areas were calculated and summed up, which is the contour area of the bilayer surface.

## 3. Results and Discussion

### 3.1. Effects of Cholesterol and MSPC on the Bilayer Conformation, Dynamics, and Curvature

**Figure 2:**
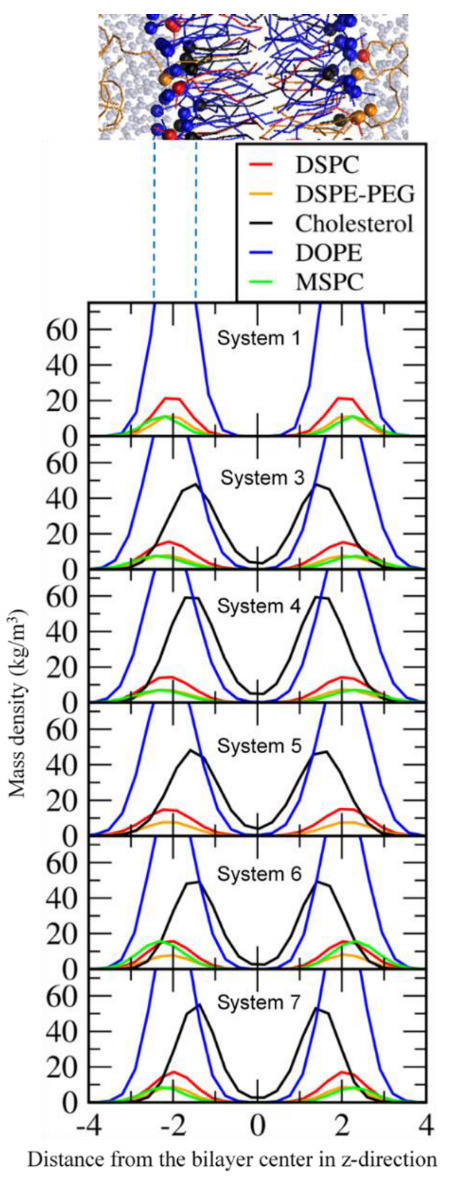
Mass density profiles for the phosphate groups of lipids and the hydroxyl group of cholesterol.

Bilayers composed of DSPC, DSPE-PEG, DOPE (or DOPC), MSPC, and cholesterol were simulated at different ratios of MSPC and cholesterol (Table 1). To quantify bilayer conformations, mass densities for the phosphate groups of DSPC, DSPE-PEG, DOPE (or DOPC), MSPC, and the hydroxyl group of cholesterol were calculated. Figure 2 shows that headgroups of DSPC, DSPE-PEG, DOPE (or DOPC), and MSPC are positioned in the bilayer-surface region, indicating stable bilayer formation. In particular, the hydroxyl group of cholesterol are positioned between lipid headgroup and tail regions, while the headgroups of MSPC, which is a lyso-lipid having a single hydrocarbon tail, are more slightly toward the water region than are those of other lipids, indicating that large headgroups of the inverted cone-shaped MSPC (lyso-lipid) tend to occupy the vacancy in the bilayer surface consisting of small headgroups of the cone-shaped DOPE. 

**Figure 3:**
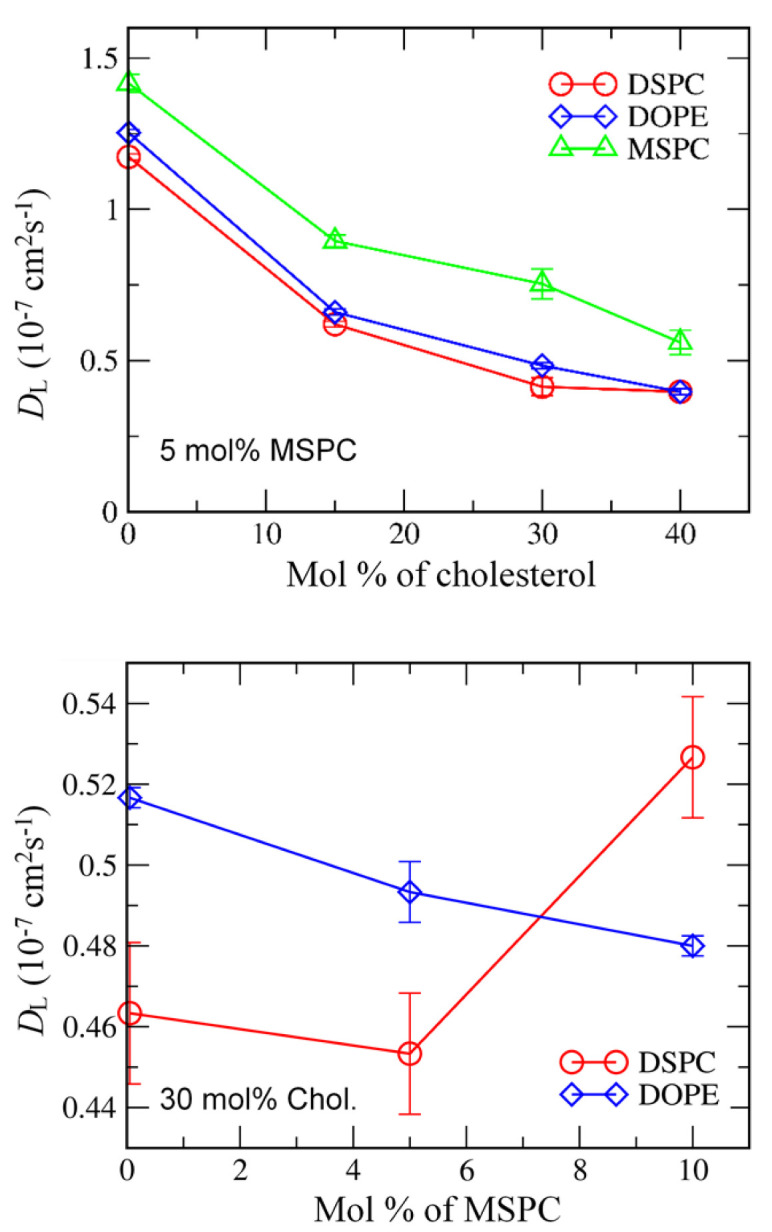
Lateral diffusion coefficients (*D*_L_) of lipids as functions of cholesterol (**top**) and MSPC (**bottom**) concentrations.

To understand the effect of cholesterol and MSPC on the bilayer dynamics, lateral diffusion coefficients of DSPC, DOPE, and MSPC lipids were calculated from the slopes of the mean-square displacements of lipid-phosphate and cholesterol-hydroxyl groups in the xy-plane (the direction perpendicular to the bilayer normal). In Figure 3, lateral diffusivities of DSPC, DOPE, and MSPC decrease as the concentration of cholesterol increases, which indicates that cholesterol-lipid interactions restrict the motion of lipids and thus reduce lateral dynamics of bilayers, leading to an increase in the bilayer stability, in agreement with previous simulations and experiments [58,59,60,61]. Although diversity of lipid components and ratios in mixture membranes preclude any quantitative comparison (such as area per lipid and lateral diffusion) between simulations and experiments, this packing effect of cholesterol on membrane structure, dynamics, and phase behavior is qualitatively consistent with the previous theoretical study [62], experiment [63] and simulation [64]. As the concentration of MSPC increases, lateral diffusivities of DOPE decrease, whereas those of DSPC increase, indicating that the inverted cone-shaped MSPC lipids tend to interact with the cone-shaped DOPE lipids rather than with the cylindrical-shaped DSPC lipids and thus reduce the lateral dynamics of DOPE.

**Figure 4:**
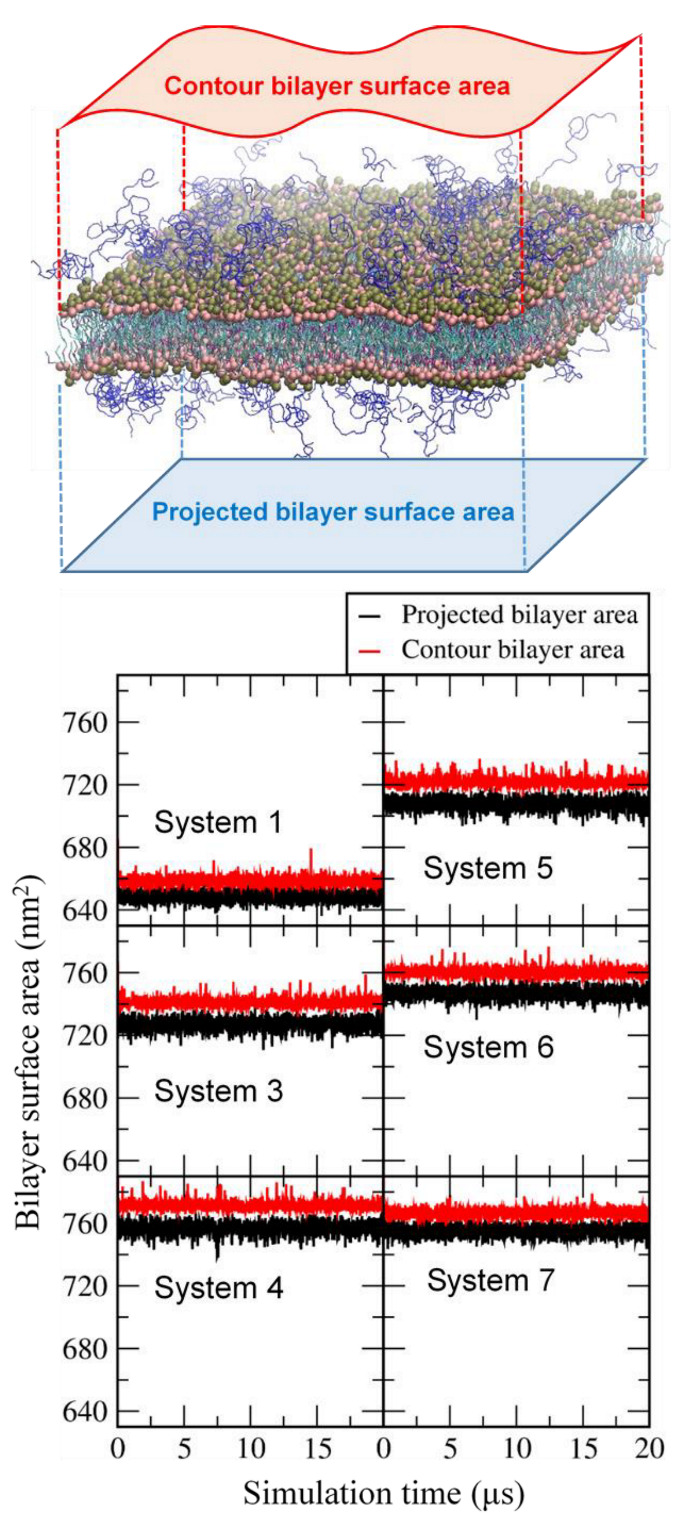
Projected and contour bilayer-surface areas as a function of simulation time.

**Figure 5:**
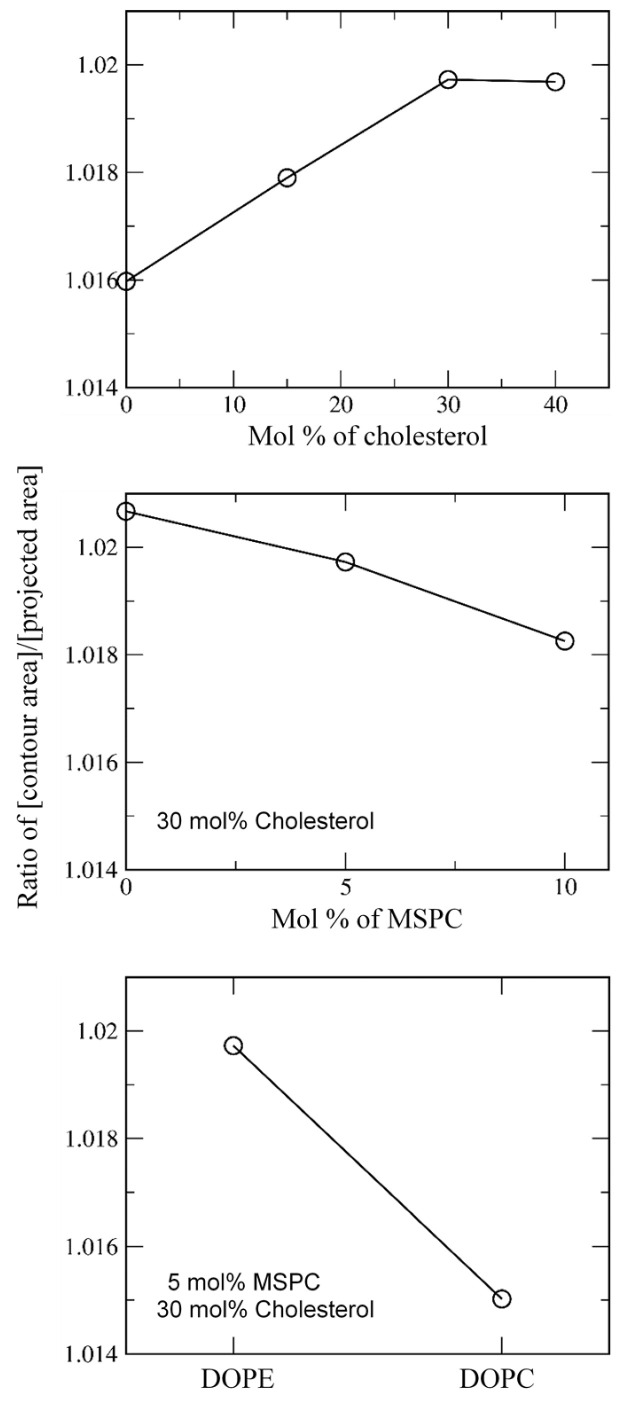
The ratio of [contour bilayer-surface area]/[projected bilayer-surface area] as functions of cholesterol (**top**), MSPC (**middle**), and lipid type (DOPE vs. DOPC; **bottom**).

To further understand the effect of cholesterol and MSPC on the bilayer stability, projected and contour areas of bilayer surfaces were calculated in the xy-dimension. In Figure 4, surface areas reach steady-state values within 5 μs, showing that bilayers are well equilibrated within the simulated timescale. For all bilayers, contour areas are slightly larger than projected areas (system sizes), as expected, because of bilayer curvature. To compare the effects of cholesterol and MSPC on bilayer curvature, the ratios of contour areas to projected areas were calculated. In Figure 5, the ratio values increase with increasing the cholesterol concentration or decreasing the MSPC concentration, which indicate that the bilayer curvature can be increased by cholesterol but decreased by MSPC, showing the opposite effects of cholesterol and MSPC on the bilayer curvature. The ratio is higher for the bilayer with DOPE than for the bilayer with DOPC, showing the higher curvature for DOPE than for DOPC, apparently because a cone-shaped DOPE has a smaller head group than does cylindrical-shaped DOPC and thus can induce negative curvature in the bilayer.

**Figure 6:**
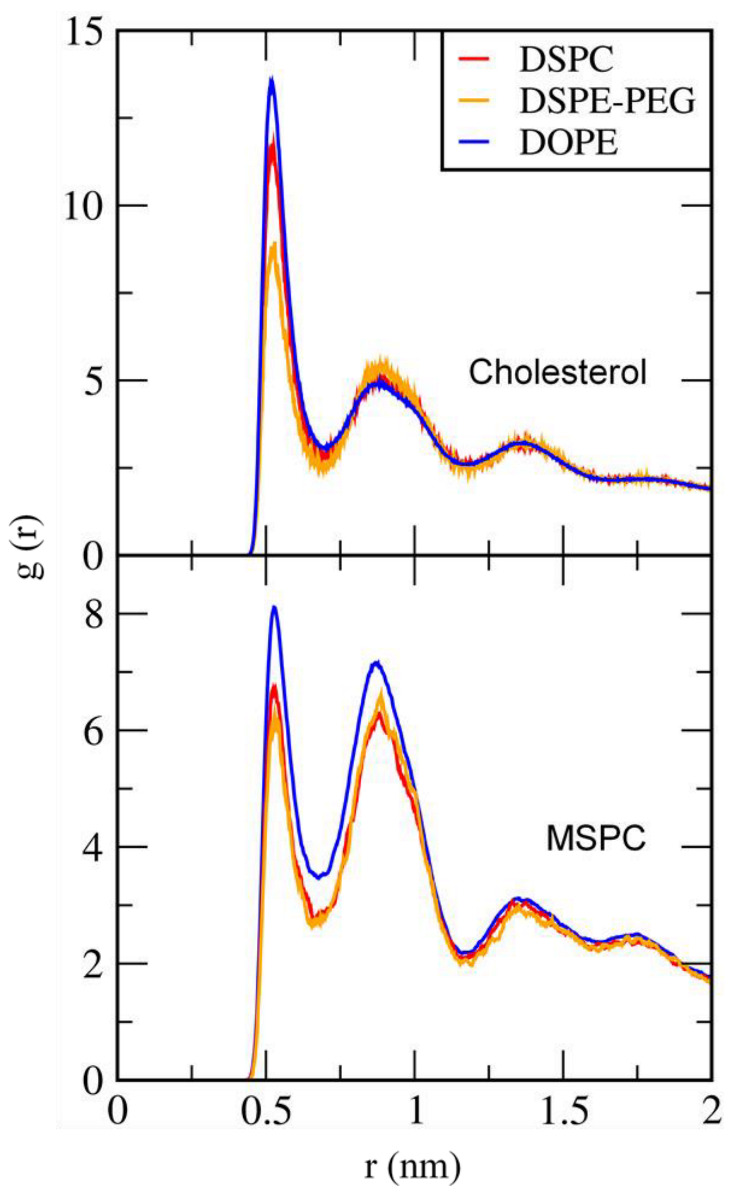
Radial distribution functions (RDFs) of DSPC, DSPE-PEG, and DOPE with respect to cholesterol (**top**) and MSPC (**bottom**).

To compare cholesterol-lipid and MSPC-lipid interactions, radial distribution functions (RDFs) of DSPC, DSPE-PEG, and DOPE were calculated with respect to cholesterol and MSPC. In Figure 6, there are sharp peaks for both cholesterol and MSPC, showing that both cholesterol and MSPC interact with DSPC, DSPE-PEG, and DOPE. In particular, cholesterol and MSPC show higher peaks for DOPE than for DSPE-PEG and DSPC, and this tendency is more prominently observed for MSPC than for cholesterol, although these RDF peaks do not significantly differ presumably because of an artificial effect of the simplification introduced by the CG model; this indicates that MSPC lipids tend to interact with DOPE lipids rather than with DSPC and DSPE-PEG lipids, presumably because the inverted-cone shaped MSPC can effectively pack the vacancy in the bilayer surface composed of DOPE that has the cone shape with a small headgroup.

These results, combined with Figure 2, Figure 3, Figure 4 and Figure 5, show that cholesterol molecules strongly interact with neighboring lipids and thus decrease lateral dynamics of bilayers and increase bilayer stability, while they also promote bilayer curvature and thus decrease bilayer stability, which indicate the opposite effects of cholesterol on bilayer stability, implying that cholesterol do not significantly influence bilayer stability. Likewise, MSPC strongly interact with neighboring lipids and thus decrease lateral dynamics of bilayers and increase bilayer stability, but also MSPC, which is an inverted-cone shaped lyso-lipid with a large headgroup, can effectively occupy the vacancy in the bilayer surface composed of DOPE that has a cone shape with the small headgroup, which can suppress bilayer curvature and increase bilayer stability. These simulation findings on the opposite effects of cholesterol and MSPC on bilayer stability explain experimental observations regarding that liposomes are more effectively stabilized by adding MSPC than by adding cholesterol [38]. Our simulations also show that the bilayer with the cylindrical-shaped DOPC is more stable than the bilayer with the cone-shaped DOPE, which supports experimental results showing that ultrasound can promote little release of doxorubicin from liposomes composed of DOPC [38].

### 3.2. Effect of Lipid Shape on the Bilayer Conformation, Dynamics, and Curvature

Experimentally, Kim et al. showed that the high concentration of MSPC does not only increase liposome stability but also can prevent liposomes from releasing doxorubicin because of the increased stability of liposome [38]. To resolve this, they found that the optimal ratio of DSPC, DSPE-PEG, cholesterol, DOPE, and DOPC is 10:5:30:65:5, which significantly stabilizes liposomes but also allows drug molecules to be released from liposomes upon applying ultrasound [39]. The liposome with this specific ratio of lipids and cholesterol has been named “IMP301” in experiments [39], which we will also use in this work (system 3 in Table 1). Experiments showed the higher level of ultrasound-induced doxorubicin leakage from IMP301 than from the commercial liposome “DOXIL” composed of hydro Soy PC (HSPC), cholesterol, and DSPE-PEG at their ratios of 56:38:5, indicating great potential of IMP301 for drug delivery applications [39]. To compare membrane stability of IMP301 and DOXIL, DOXIL bilayer was also simulated at the same conditions as IMP301. 

**Figure 7:**
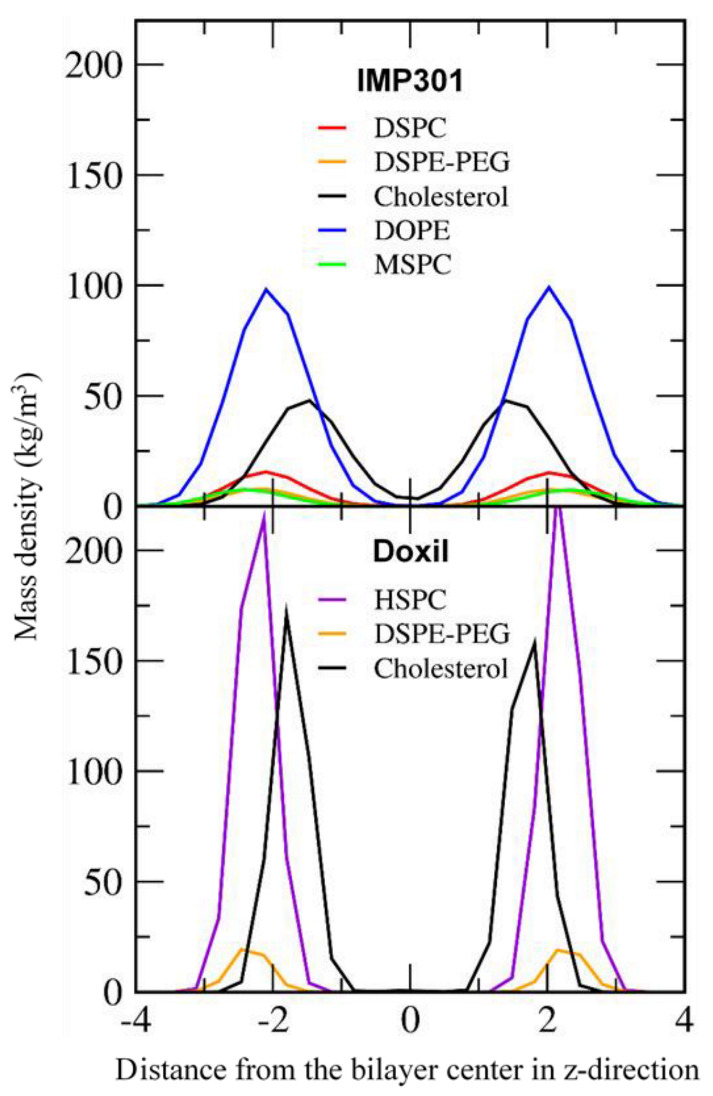
Mass density profiles for the phosphate group of lipids and the hydroxyl group of cholesterol for IMP301 and DOXIL.

**Figure 8:**
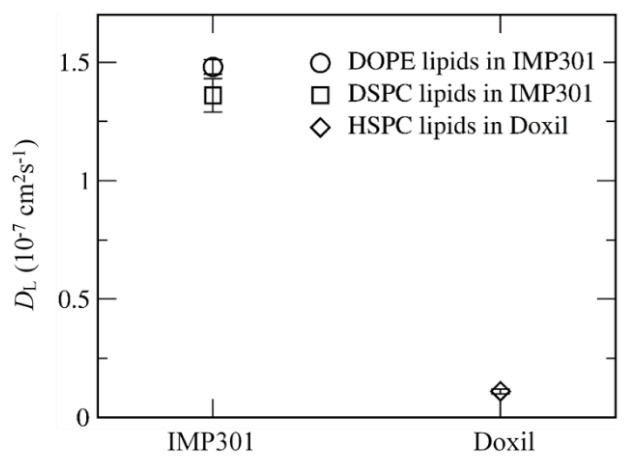
Lateral diffusion coefficients (*D*_L_) of lipids for IMP301 and DOXIL bilayers.

Figure 7 compares mass densities of IMP301 and DOXIL bilayers, showing the thicker bilayer for DOXIL than for IMP301. In Figure 8, lateral diffusivities of IMP301 and DOXIL bilayers are respectively 1.36~1.48 and 0.11 ± 0.01 (×10^−7^ cm^2^/s), showing a much higher diffusivity of IMP301. These results indicate that DOXIL bilayer is more ordered and thicker than IMP301 bilayer and thus relatively more stable. Note that because HSPC (16:0, 18:0) includes a longer hydrocarbon tail than DPPC (16:0, 16:0) does, the phase-transition temperature of HSPC should be higher than that of DPPC, indicating that HSPC bilayer forms the ordered-gel phase at this simulated temperature. 

**Figure 9:**
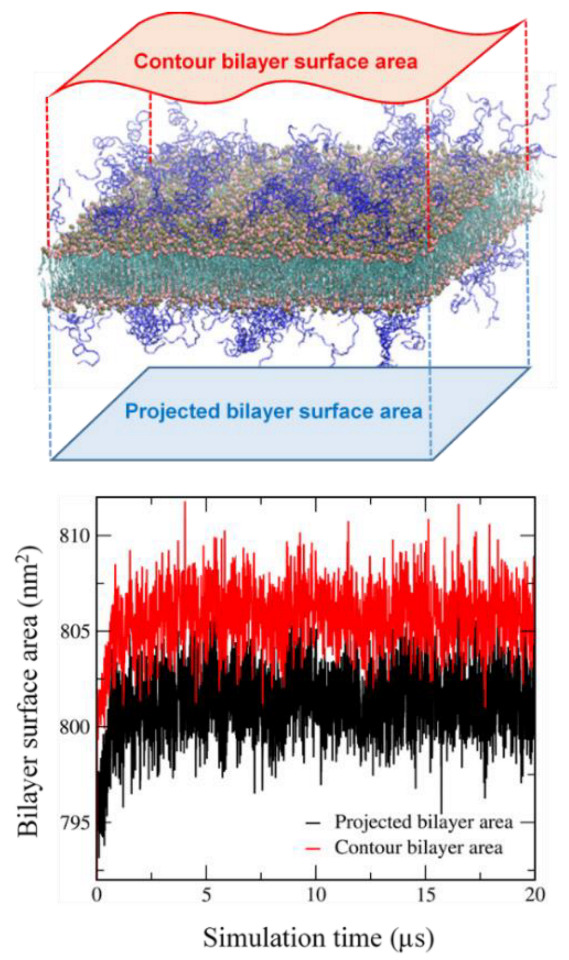
Projected and contour bilayer-surface areas of DOXIL as a function of simulation time.

**Figure 10:**
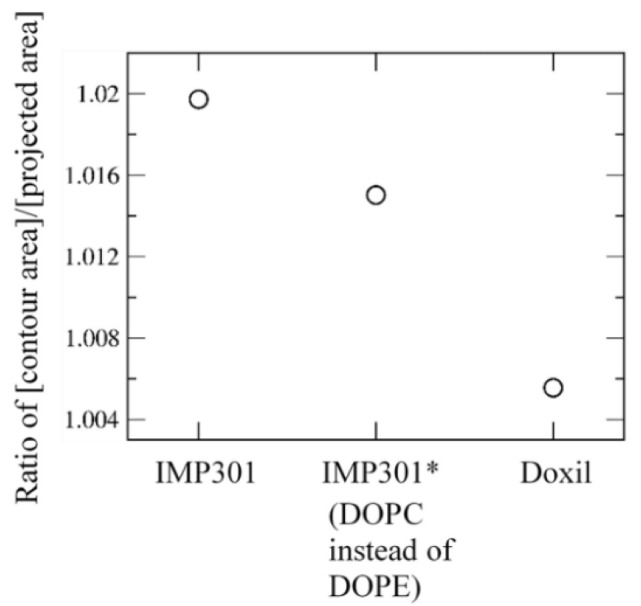
The ratio of [contour bilayer-surface area]/[projected bilayer-surface area] for IMP301, IMP301* (including DOPC instead of DOPE), and DOXIL.

To compare the extents of curvature for IMP301 and DOXIL bilayers, contour and projected bilayer-surface areas of DOXIL were calculated. In Figure 9, surface areas reach steady-state values within 5 μs, showing that bilayers are well equilibrated. Contour areas are slightly larger than projected areas, indicating the presence of bilayer curvature, as also observed for IMP301 in Figure 4; however, Figure 10 shows that the ratio of the contour area to the projected area is higher for IMP301 than for DOXIL, indicating the higher curvature for IMP301 than for DOXIL. Recall from Figure 5 that curvature is lower for the bilayer with DOPC than the bilayer with DOPE. In Figure 10, the curvature of DOXIL bilayer is even lower than that of DOPC bilayer. These results imply that the DOXIL bilayer mainly consists of cylindrical-shaped HSPC lipids and thus induce less curvature and higher stability than do the bilayers with cone-shaped DOPE lipids (IMP301) or DOPC lipids (IMP301*), which supports experimental results showing the higher extent of ultrasound-responsive drug-release from IMP301 than from DOXIL [39]. 

**Figure 11:**
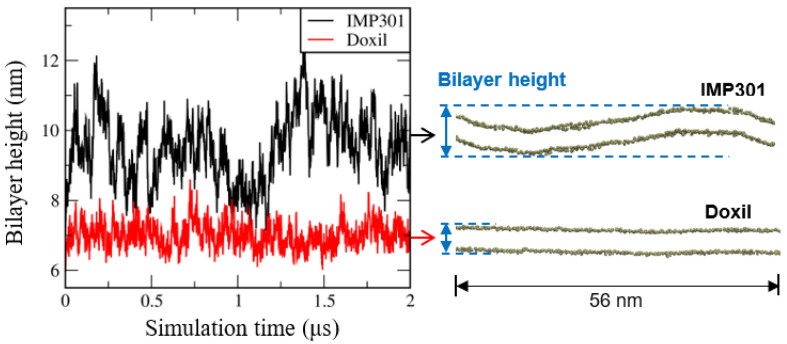
Bilayer height, defined as the maximum distance between phosphates projected along the bilayer normal, of 56 nm-sized IMP301 and Doxil bilayers as a function of simulation time (**left**) and their snapshots of the side view (**right**).

To confirm the formation of bilayer curvature, much larger (56 nm-sized) bilayers of IMP301 and Doxil were also simulated for 2 µs. Figure 11 shows side-view snapshots of bilayers and the bilayer height as a function of simulation time, indicating the formation of bilayer curvature for IMP301 but not for Doxil. In particular, bilayer heights, defined as the maximum distance between phosphates projected along the bilayer normal, are much larger for IMP301 than for Doxil, clearly showing larger membrane curvature for IMP301 than for Doxil, consistent with results from Figure 9 and Figure 10.

**Figure 12:**
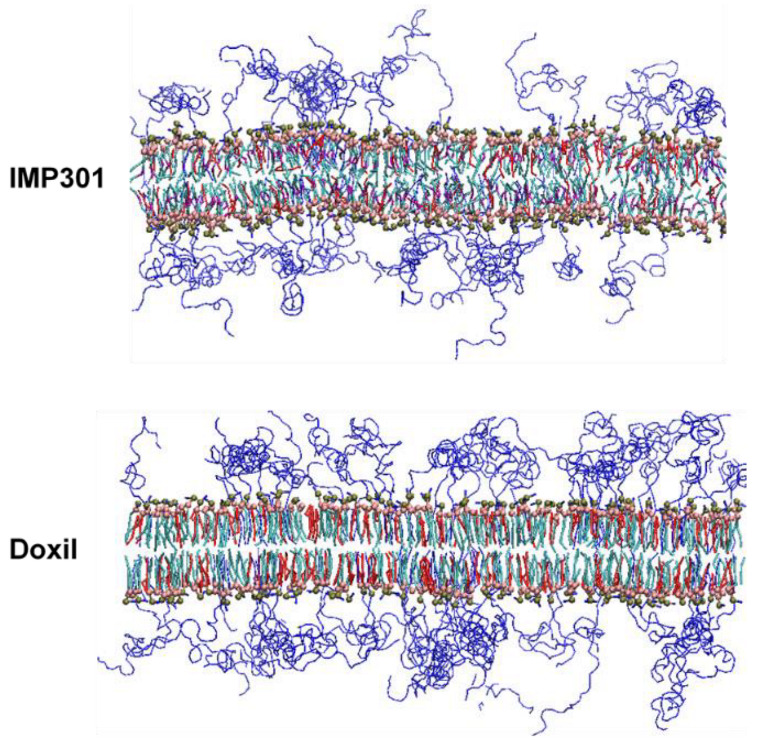
Side views of final configurations for IMP301 and DOXIL bilayers. Lipid headgroups and tails are respectively represented as brown (phosphate)/pink (glycerol) dots and light-blue lines, while cholesterol and PEG are colored in red and blue lines, respectively. For clarity, water and counterion molecules are omitted.

Experimentally, Kim et al. optimized the concentration of MSPC [38] and developed the ultrasound-responsive drug-loaded liposome, IMP301, that does not only retain its membrane stability but also have the high drug-release efficacy upon applying ultrasound, showing great potential for drug delivery applications [39]. Our simulation results show that the presence of cholesterol can increase bilayer stability but also induce bilayer curvature that reduces bilayer stability, indicating that two opposite effects of cholesterol evenly occur and thus do not significantly influence bilayer stability. In contrast, the presence of MSPC only decreases bilayer dynamics, leading to an increase in bilayer stability. Simulations also show that IMP301 bilayer has faster lateral dynamics and higher curvature than DOXIL bilayer does, indicating the more disordered phase for IMP301 bilayer than for DOXIL bilayer as visualized in Figure 12, presumably because cone-shaped DOPE lipids (IMP301) can induce more bilayer curvature than do cylindrical-shaped HSPC lipids (DOXIL).

### 3.3. Pore Formation Induced by an External Pressure

Experimental studies have shown that ultrasound can be applied to trigger the drug release from liposomes, which has been interpreted as an indication that the cone-shaped DOPE lipids induce negative bilayer curvature, leading to the formation of small pores that can release drug molecules [65]. To understand this, IMP301 bilayer was simulated with an external pressure of 100 bar applied in the direction of the bilayer normal, which mimics a pressure of ~100 bar produced by ultrasound in the experiment. Note that the extent of ultrasound is controlled by many parameters such as pressure amplitude, frequency, burst length, and pulse repetition frequency. These differ from the external pressure simply applied in our simulations, which allows only qualitative comparison between experiment and simulation. 

**Figure 13:**
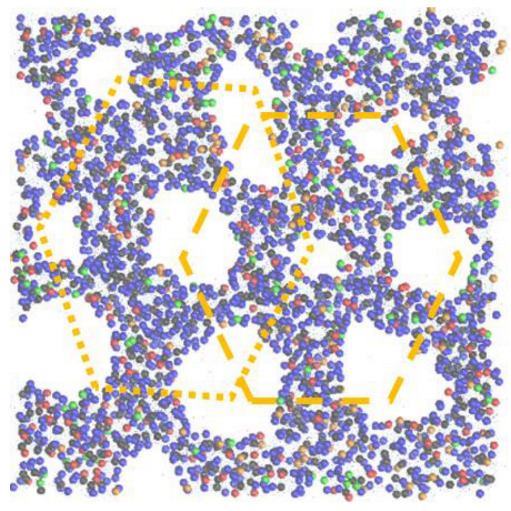
A snapshot for the top view of IMP301 bilayer at the end of simulation. Pore arrangement is highlighted as yellow hexagons. Phosphate beads of DSPC, DSPE-PEG, DOPE, MSPC, and hydroxyl beads of cholesterol are presented as thick dots colored in red, orange, blue, green, and black, respectively.

Figure 13 shows that pore formation occurs upon applying an external pressure of 100 bar in the direction of bilayer normal. Water molecules can penetrate through these pores that are surrounded by hydrophilic lipid headgroups. In particular, pores form the hexagonal arrangement, similar to the experimental suggestion of a hexagonal phase in the bilayer mainly composed of DOPE [65], although this hexagonal arrangement has an irregular shape, presumably because of a mixture of various lipids.

**Figure 14:**
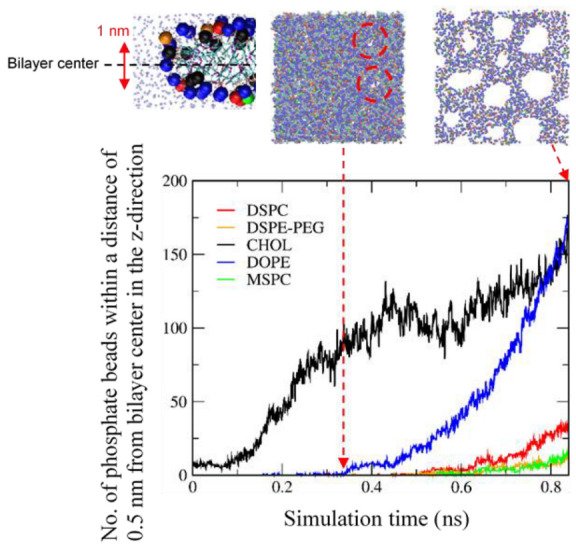
Number of beads of lipid phosphates or cholesterol hydroxyl-groups within a distance of 0.5 nm from bilayer center in the z direction, as a function of simulation time.

**Figure 15:**
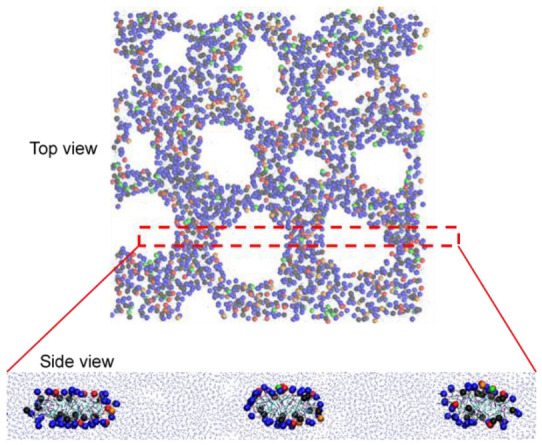
Top and side views at the end of simulation. Phosphate beads of DSPC, DSPE-PEG, DOPE, MSPC, and hydroxyl beads of cholesterol are represented as thick dots colored in red, orange, blue, green, and black, respectively.

To understand the pore structure, we calculated the numbers of lipids and cholesterol near the pore edge. The xy-plane parallel to the bilayer surface was equally divided into 49 voxels by using a grid (7 × 7 grid). For each voxel, the z component (normal to the bilayer) of the center of mass (COM) of lipid phosphates was averaged over both leaflets of the bilayer, and the z coordinates of this was taken to be the bilayer center for that voxel. Lipid-phosphate or cholesterol-hydroxyl beads “around the bilayer center” are taken to be those that are within 0.5 nm in the z direction from the bilayer center from the voxel in which the bead is located. Figure 14 shows that cholesterol molecules are close to the bilayer center for whole simulation time, while other lipids become close to the bilayer center at the simulation time of ~0.33 ns, at which pores begin to form, indicating an increase in the amount of lipid headgroups near the pore edge. DOPE lipids, which are the major component (65%) of the bilayer, are also predominantly distributed at the bilayer edge. In Figure 15, the side view of the bilayer edge and pore shows that the bilayer edge does not only consist of DOPE but also include DSPC, DSPE-PEG, MSPC, and cholesterol, implying that MSPC and cholesterol, which have the relatively large headgroup, are positioned in the vacancy between small headgroups of cone-shaped DOPE lipids and thus help to retain the curvature of the pore edge. Note that differences of mass transport conditions and durations of the simulations compared to those in experiments preclude any quantitative comparison between the two. Moreover, the ultrasound experiment must be controlled by many parameters such as pressure amplitude, frequency, burst length, and pulse repetition frequency, which cannot be mimicked by applying a constant external pressure; however, we have clearly shown different effects of various lipids and cholesterol on membrane conformation, dynamics, and curvature, which helps to explain experimental observations showing that MSPC lipids more significantly influence liposome stability than cholesterol does [38], and that IMP301 more effectively releases drug molecules by applying ultrasound than DOXIL does [39]. 

## 4. Conclusions

We performed CG MD simulations of bilayers composed of various lipids and cholesterol at different ratios. The lateral dynamics and curvature of bilayers decrease as the concentration of MSPC increases because inverted-cone shaped MSPC (lyso-lipids) tend to pack the vacancy in the bilayer mainly composed of cone-shaped DOPE, leading to an increase in bilayer stability. In contrast, as the cholesterol concentration increases, cholesterol-lipid interactions restrict the lateral dynamics of bilayer but also promote bilayer curvature, showing that these opposite effects of cholesterol on bilayer stability simultaneously occur. These indicate that MSPC lipids effectively increase bilayer stability, while cholesterol molecules do not significantly influence bilayer stability, in agreement with experiments. IMP301, which is the liposome experimentally optimized to achieve the high stability as well as the high drug-release rate upon applying ultrasound, and DOXIL (commercial liposome) bilayers were simulated and compared in terms of their dynamics, curvature, and lipid order, showing that IMP301 bilayer composed of cone-shaped DOPE has higher lateral dynamics and curvature than does DOXIL bilayer composed of cylindrical-shaped HSPC. These indicate that IMP301 bilayer is more disordered than DOXIL bilayer, which supports experiments showing the higher extent of drug-release rate for IMP301 liposome than for DOXIL liposome. To mimic ultrasound, a high external pressure was applied, leading to the formation of small pores that are surrounded by hydrophilic lipid headgroups and hexagonally arranged. Our findings help to explain experimental observations showing that liposomes are stabilized by MSPC rather than by cholesterol, and that the liposome with DOPE more effectively releases drug molecules upon applying ultrasound than does the liposome with HSPC.

## Figures and Tables

**Table 1:** List of simulations. Lipid ratios are represented as DSPC: DSPE-PEG: Chol.: DOPE (or DOPC): MSPC.

Simulation System No.	No. of Membrane Components						Ratio
	DSPC	DSPE-PEG	Chol.	DOPE	DOPC	MSPC	
1	256	128	-	1664	-	128	10:5:0:65:5
2	256	128	384	1664	-	128	10:5:15:65:5
3 (IMP301)	256	128	768	1664	-	128	10:5:30:65:5
4	256	128	1024	1664	-	128	10:5:40:65:5
5	256	128	768	1664	-	-	10:5:30:65:0
6	256	128	768	1664	-	256	10:5:30:65:10
7	256	128	768	-	1664	128	10:5:30:65:5
8	1920	128	768	-	-	128	75:5:30:0:5
9	1024	512	3072	6656	-	512	10:5:30:65:5

## Data Availability

Not applicable.
